# Biocompatibility of Resin-based Dental Materials

**DOI:** 10.3390/ma2020514

**Published:** 2009-04-28

**Authors:** Keyvan Moharamzadeh, Ian M. Brook, Richard Van Noort

**Affiliations:** School of Clinical Dentistry, University of Sheffield, Claremont Crescent, Sheffield S10 2TA, United Kingdom; E-Mails: i.brook@Sheffield.ac.uk (I.B.); r.vannoort@Sheffield.ac.uk (R.N.)

**Keywords:** Biocompatibility, dental materials, composite resin

## Abstract

Oral and mucosal adverse reactions to resin-based dental materials have been reported. Numerous studies have examined the biocompatibility of restorative dental materials and their components, and a wide range of test systems for the evaluation of the biological effects of these materials have been developed. This article reviews the biological aspects of resin-based dental materials and discusses the conventional as well as the new techniques used for biocompatibility assessment of dental materials.

## 1. Introduction

Biocompatibility is defined as “the ability of a material to function in a specific application in the presence of an appropriate host response” [1]. This definition implies an interaction among a host, a material, and an expected function of the material. All three factors must be in harmony before the material can be considered biocompatible [2]. Since the principal intended action of most dental materials is achieved by their physical/mechanical properties, the expression ‘appropriate host response’ in most cases means no adverse reaction of the living system to the presence of such a material [3]. Adverse reactions to various dental materials have been reported [4]. Although these reactions are rare, considering the millions of treatments provided, many individuals may potentially be affected [5].

In some cases, the dental staff is at higher risk of adverse reactions to biomaterials than the patients. For example dental resins (primarily acrylics) and rubber products cause adverse reactions mainly in dental staff. Hand and fingertip reactions such as dry, cracking and flaking skin, itching, irritation, and swelling have been reported [4] as well as generalized neuropathy after 14 years exposure to methacrylates [6]. Reactions to many types of prosthodontic materials can be severe, career-threatening, and even life threatening in rare instances [7].

In recent years the use of resin-based restorative materials has increased in dentistry because of better aesthetics, improved adhesion to enamel and dentine, and worries about adverse effects of mercury from amalgam. It has been shown that components of dental composite resins can be released from the materials [8,9,10,11,12,13]. Unpolimerized monomers can be leached into saliva [14,15,16] and cause adverse reactions [7]. According to a national survey of adverse reactions to dental materials in the UK, dental resins are the main cause of adverse reactions in dental technicians, and more than 12% of adverse reactions in patients are related to resin-based dental materials [4]. These reactions in patients can be classified into two main categories: (1) local reactions and (2) systemic reactions.

## 2. Local Adverse Reactions and Evaluation Systems

**Figure 1:**
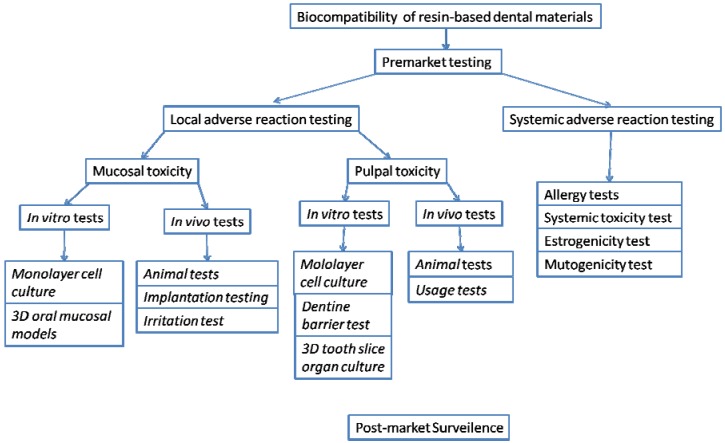
Biocompatibility tests relevant to resin-based dental materials.

Resin-based dental materials such as composite resins and denture-base materials come into direct contact with oral mucosa and can cause adverse reactions on oral mucosa. Restorative materials and dentine bonding agents can also affect the pulp due to release of leachable components through the permeable dentin. Therefore, local adverse reactions caused by resin-based materials can be assessed from two different viewpoints: (1) mucosal toxicity and (2) pulpal toxicity (Figure 1).

### 2.1. Mucosal toxicity testing

**Figure 2:**
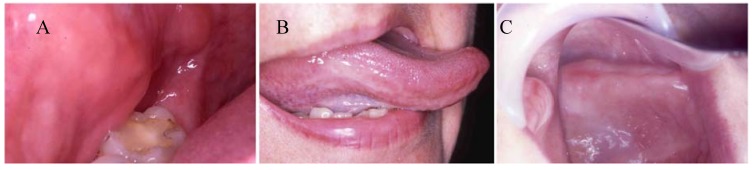
Clinical cases of adverse reactions to resin-based dental materials: (A) Oral lichenoid reaction to an occlusal composite restoration on the buccal mucosa; (B) Oral lichenoid reaction to a lingual composite restoration on the lateral border of tongue; and (C) allergic reaction to denture base material on the alveolar ridge and hard palate.

It has been reported that resin-based dental materials can cause adverse reactions on oral mucosa such as mucosal irritation, epithelial proliferation and oral lichenoid reactions (Figure 2) [7]. Biological effects of resin-based materials on oral mucosa can be assessed using two different types of biocompatibility tests: (a) *In vitro* tests and (b) *In vivo* tests.

#### 2.1.1. *In vitro* mucotoxicity tests

*In vitro* biocompatibility tests are performed outside of a living organism. The objective of *in vitro* tests is to simulate biological reactions to materials when they are placed on or into tissue of the body [17]. The effect of the material is determined by measuring the number, growth rate, metabolic function, or other cellular function of the cells exposed to the material [2]. These tests are suitable to survey new products compared to expensive and time-consuming animal tests. They are repeatable, experimentally controllable, fast and relatively simple, and there is no ethical problem. The limitations of *in vitro* tests are the lack of simulation of the *in vivo* situation, and questionable clinical relevance [3]. There are different approaches to assess the *in vitro* biocompatibility of biomaterials. All of the cytotoxicity test methods have three main parts: a biological system, a cell/material contact, and a biological endpoint.

##### Biological system

In terms of *in vitro* mucosal toxicity assessment of resin-based dental materials, the biological system can be (a) monolayer culture of oral mucosal cells or (b) a 3D tissue-engineered models of human oral mucosa.

(a) Monolayer cell culture system

In monolayer cell culture, specific cells of known type are inoculated into and maintained in the culture medium. Gingival fibroblasts and keratinocytes are appropriate for cytotoxicity testing of dental materials that are in close proximity of gingival tissue.

Human gingival fibroblasts have been frequently used to test the biocompatibility of dental materials [18,19,20,21,22,23]. Their relative merits are that they can be easily isolated from patients and can grow fast in normal culture medium. Also they show high sensitivity in cytotoxicity tests. Other cell lines that have been widely used include L-929 mouse fibroblasts [24,25,26,27,28] and 3T3 mouse fibroblasts [29,30,31,32,33,34,35]. The choice of cell type also depends on the type of biological endpoint used in cytotoxicity test. Some biological tests require specific types of cells. For instance, THP-1 monocytes and fibroblasts are suitable to study cytokine release from these cells [36,37].

Sensitivities of different cell lines to different dental materials have been investigated. It has been shown that normal human fibroblasts are more sensitive than mouse fibroblasts [38,39], and mouse macrophages are more sensitive than hamster fibroblasts BHK-21 (C-13) [40]. Geurtsen *et al.* reported that human periodontal ligament (PDL) and pulp fibroblasts were more sensitive than human gingival fibroblasts and mouse 3T3 cells [41]. Although these studies suggest that different cell lines have different sensitivity to biomaterials, the ranking of the cell lines according to their sensitivity varies with the assay technique used [42]. For example, Johnson *et al.*, who measured cellular response of 12 standardized cell lines to 20 materials to explore relative sensitivity of *in vitro* biocompatibility systems, reported that established cell lines derived from different tissues differ especially in sensitivity, depending on the specific test method used [43]. In another study they reported that assays using established cell lines were more reproducible than assays using primary cells [44]. In addition to the batch-to-batch variations using monolayer cell cultures, as mentioned earlier, the main limitation of the monolayer systems is the lack of clinical relevance. The results obtained from monolayer cell culture systems may not apply to normal human oral mucosa and cannot be extrapolated to the patients since epithelial cells in monolayer culture lack the differentiated function and barrier properties of cells found in normal human oral mucosa. In order to obtain an accurate risk assessment and simulate the clinical situation as closely as possible, 3D tissue engineered models of oral mucosa have been introduced.

(b) Three-dimensional tissue engineered models

In recent years three-dimensional tissue engineered models of human oral mucosa have been developed for *in vitro* biocompatibility assessment of resin-based dental materials. [45,46,47]. Schmalz *et al.* introduced a three-dimensional human fibroblast and keratinocyte co-culture system on a nylon mesh to assess mucosal irritancy of metals used in dentistry [48]. They also used another test system that consisted of three-dimensional fibroblast culture on nylon mesh in an *in vitro* pulp chamber [49]. Schuster *et al.* used three-dimensional culture of transfected bovine pulp-derived cells on polyamide meshes to test cytotoxicity of dental materials [50].

These *in vitro* models seem promising for biocompatibility evaluation of dental biomaterials since they reflect the clinical situation better than single layer cell culture test models and they allow multiple-endpoint analysis of the response of oral mucosa to restorative materials. Therefore, they can reduce the need for animal testing and be more specific.

##### Cell/material contact

Adequate contact between cells and test material is very important in biological evaluation of materials. Contact between cells and material can be achieved in three ways: direct contact, indirect contact, and contact through extracts [51].

(a) Direct contact

In a test based on direct contact the material is in physical contact with the cells or the culture medium. Water-soluble materials are directly dissolved in the culture medium and there is good cell/material contact and high sensitivity [51].

Direct cell/material contact for non-water-soluble materials can be established in the following ways:

1. The test specimen is placed as close to the tissue explant as possible [52].
2. The test specimen is placed on top of an established cell monolayer [24,27,38,53]
3. The test specimen is placed on the bottom of a culture vessel, a cell suspension is added and a cell monolayer is allowed to establish around the specimen [54].
4. The cells are cultured directly on the specimens [54,55].

A good cell/material contact may be obtained by growing the cells directly on the specimen. However, in this case the surface characteristics of the material is important because if the material has low surface energy, the cells will not adhere to the surface of the material and consequently they will not grow well [51].

Kasten *et al*. introduced a model experimental culture system to screen polymerized dental materials for diffusible toxic products. The system employed human gingival fibroblast culture in plates containing immobilized specimens of polymerized resins. Cytotoxicity of the materials was assessed by measuring cell death as a function of time of exposure and distance from the sample [56].

In all of the above methods the test specimen is covered by the culture medium. This may affect the test results as the culture medium can mitigate the toxic effects of the material in several ways, for instance, by diluting the leachable components or by binding of toxic monomers to proteins present in serum-containing culture medium [12].

Using 3D tissue engineered oral mucosal models it is possible to expose the surface of the epithelium to test materials in a direct mucosal contact format and minimize the effect of the culture medium on the specimen as the tissue is fed only from the connective tissue side. This arrangement is very similar to what happens in the clinical situation.

(b) Indirect contact

In a test system based on indirect cell/material contact, the specimen is separated from the cells by a permeable intermediate. This method is independent of the physical state of the material and can be used with solid, semi-solid, and liquid materials and since the test specimen is not covered by culture medium, the materials can be tested in an unset state [51]. The first indirect cell/material contact called the agar overlay technique was introduced by Guess *et al.* [57] and is designed to evaluate the cytotoxic effects of diffused leachable components through an agar layer covering a monolayer cell culture. This technique has been frequently used in biocompatibility testing of dental materials [25,58,59,60].

Another indirect cell/material contact method was used by Tyas [61], in which a synthetic filter or dentine slice was used as an intermediate between the specimen and culture medium. Wennberg *et al.* [62] used cells cultured on Millipore filters as an indirect test method. Since the filter is thin and cell processes often grow into the pores, there is an intimate contact between the cells and the test material and even non-water-soluble leachable components can reach the cells.

(c) Contact through extracts and elutes

Contact between insoluble materials and the cells can be established by using an emulsifying agent or extracting leachable components by a solvent.

In some cytotoxicity studies dental resin monomers have been dissolved in dimethyl sulphoxide (DMSO) or ethanol and diluted with culture medium [23,28,36,41,63,64,65]. Concentrations of DMSO or ethanol that have been used in these studies were below the minimum concentration required to produce a cytotoxic response and a control group of DMSO or ethanol with maximum concentration in culture medium is used for accurate evaluation of the monomer cytotoxicity.

Extraction technique has been frequently used in cytotoxicity evaluation of different dental materials such as restorative materials [32,66,67,68], dental cements [38], amalgams [69], denture base resins [70], and dentine adhesives [20].

Different extraction media have been used such as: culture medium [20,34,66,67,70], distilled water [33,71], saline [38], balanced salt solution [72], and acetone plus ethanol in saline [73].

Studies that compare different extraction techniques are rare. Hanks *et al.* compared saline and culture medium as extracting media. In their experiment the saline extract was cytotoxic but medium extract was not [38]. It has been shown that the type of extraction media and the time of analysis have a significant effect on the detection of monomer released from experimental composite resins into various aqueous media. This may lead to false-negative results in cytotoxicity testing of dental materials [12].

##### Biological endpoint

In cytotoxicity tests the cell reaction can be described morphologically or quantitatively based on cell viability, proliferation and cell function such as apoptosis, adhesion, migration, and secretion of certain substances. In the following paragraphs some of the most widely used assays in biocompatibility testing of dental materials are discussed.

(a) Morphological assessment

This method is based on pathological changes in the cells such as nuclear enlargement, binucleation, nuclear anomalities, and vacuoles. Dead cells are characterized by nuclear disintegration, and pyknoses. Using a 3D human oral mucosal model it was possible to visualize the direct damage caused by resin monomers to different layers of oral mucosa [47]. However, there is not much data available on how the degree of the damage caused by resin monomers on 3D *in vitro* oral mucosal model would compare to that of normal human oral mucosa. Therefore, there is need for clinical validation of these tissue engineered oral mucosal models for biological assessment of resin-based restorative dental materials.

(b) Cell viability and proliferation assays

In clinical situation, damaged part of oral mucosa has a lower metabolic rate and proliferation status than healthy oral mucosa due to low number of viable cells present in the damaged tissue. There are a number of different assays that can be used to measure the viability and proliferation status of the cells exposed to test materials *in vitro* to assess the materials relative toxicity.

##### MTT assay

The colorimetric MTT [(3-(4,5-dmethylthiazol-2-yl)-2,5-diphenyltetrazolium bromide] assay, developed by Mossman, indicates the effects on cell viability by alterations of mitochondrial dehydrogenase activities. It is based on the conversion of the water-soluble methylthiazole tetrazolium to an insoluble purple formazan. This formazan is then solubilized, and its concentration can be determined spectrophotometrically [74]. The MTT assay is the most common test to evaluate the cytotoxicity of dental materials [23,34,41,50,67,68,75,76] because it is a rapid and inexpensive method.

##### Alamar blue assay

Alamar blue is a safe, non-toxic aqueous dye that is used to evaluate cell viability and cell proliferation [77]. The Alamar blue assay incorporates a fluorometric/colorimetric growth indicator based on detection of metabolic activity. The system incorporates an oxidation-reduction indicator that both fluoresces and changes color in response to chemical reduction of growth medium resulting from cell growth [78]. Alamar blue is soluble, stable in culture medium and is non- toxic. Therefore it is possible to continuously monitor the cells in culture. Specifically it does not alter the viability of cells cultured with time [79].

Alamar blue has two advantages over the MTT assay: First its change in color can be detected both spectrophotometrically and fluorometrically which is more accurate. Second, since it is not toxic to the cells it is possible to assess cell viability on more than one occasion. However, since Alamar blue assay is more expensive than the MTT assay most researchers prefer to use the MTT. Uo *et al.* has used Alamar blue assay to assess the biocompatibility of dental ceramics [80]. The Alamar blue assay has also been used in several studies for biological evaluation of resin-based dental materials using both monolayer and 3D cultures of human oral epithelial cells [47,65].

##### Neutral red assay

Neutral red is a vital dye, which is stored in viable cells and released into the surrounding medium after membrane damage and provides an index of cell viability. In the neutral red assay the fraction of surviving cells is determined by their content of the dye which is retained only by live cells and can be quantitated photometrically after controlled lysis [81]. This assay has been frequently used in cytotoxicity testing of dental materials [82,83,84,85,86,87].

##### Propidium iodide assay

This assay is based on the exclusion of a solution of propidium iodide dye (PI). If the cell membrane is damaged by any toxic substance, PI enters the cells and intercalates with the DNA and RNA [88]. The number of cells stained by the fluorescent dye can be determined by flow cytometry. As the PI is an exclusion dye, the proportion of fluorescent cells means the number of dead cells. Some studies have used this method to evaluate the cytotoxic effects of dental materials on cell cultures [83,89].

##### LDH assay

This colorimetric cytotoxicity assay measures lactate dehydrogenase (LDH), a relatively stable cytosolic enzyme that is released by cells when they undergo significant membrane damage or cytolysis. The amount of LDH released is proportional to the number of cells damaged/lysed [90]. In cytotoxicity studies, the percentage release of LDH from the material- exposed cells is calculated by comparing it to the maximum release of LDH achieved by controlled lysis of the cells [23,91,92].

##### Bromodeoxyuridine incorporation assay

By incorporation of 5-bromo-2’-deoxyuridine (BrdU) into newly synthesized DNA during cell division it is possible to detect rapidly proliferating cells with fluorescently labeled anti-BrdU antibodies or certain nucleic acid stains [93]. This assay was used by Theilig *et al*. to evaluate the effects of dental resin monomers on proliferation of human fibroblasts and keratinocytes [63].

##### 3H-thymidine incorporation assay

Another method to assess cell proliferation is to incorporate ^3^H-thymidine into cells during proliferation and detect newly synthesized DNA by radioactivity measurement. Aronson *et al.* evaluated the effect of the constituents of dental composite resins on the proliferation of human and rat mononuclear cells using ^3^H-thymidine incorporation assay [94].

##### DNA content measurement

Using DNA-intercalating dyes it is possible to determine the DNA content of the cells by measuring fluorescence intensity. The nucleic acid stains most frequently used for cell-cycle analysis are Hoechst 33,258, Hoechst 33,342 and DAPI that bind to the minor groove of DNA at AT-rich sequences [95]. Leyhausen *et al*. used this method to assess the biocompatibility of different glass ionomer cements [96].

##### Protein content measurement

Lowry *et al.* introduced a method for protein determination [97] and later Ohnishi *et al.* simplified their method [98]. Currently modifications of their methods are used for protein content measurement in cytotoxicity experiments. Reichl *et al*. used protein determination to evaluate cytotoxicity of dental composite components and mercury compounds [91].

(a) Assays based on cell function

##### Inflammatory mediators measurement

Measurement of the amount of pro-inflammatory mediators in cell culture supernatants of the cells exposed to resin-based materials is a sensitive and efficient approach that may show a direct biochemical link between the parameters measured *in vitro* and clinical effects such as inflammation *in vivo*.

In the recent decade some studies have concentrated on the influence of dental materials and their components on inflammatory markers. For example Noda *et al.* showed that sublethal exposure to triethylene glycol dimethacrylate (TEGDMA), and Hydroxyethyl methacrylate (HEMA) for two weeks alters tumor necrosis factor-alpha (TNF-α) secretion by THP-1 monocytes [36]. In another study Schmalz *et al*. demonstrated that molecules important in the initiation of inflammation like PGE_2_ or IL-6 and IL-8 were released from human oral tissue culture models after exposure to compounds of dental materials [37]. Heil *et al.* compared the sensitivity of human peripheral blood monocytes and THP-1 monocytes to resin monomers in terms of TNF-α secretion, and showed that THP-1 monocytes were more sensitive [99]. In these studies the response of the cells to the materials with and without lipopolysaccharide (LPS) stimulation has been evaluated by measuring TNF- secretion from the cells by enzyme-linked immunosorbant assay (ELISA). It has been demonstrated that exposure to high TEGDMA-containing experimental composite resins significantly increased the amount of Interlukin-1 beta (IL-1β) released from 3D tissue engineered human oral mucosal models [47].

##### Glutathione determination

Glutathione (GSH), a tripeptide, is an important intercellular reducing agent that participates in several decisive metabolic reactions and plays a crucial role in detoxification and inactivation of toxic substances such as free radicals, oxidants, and electrophiles [100].

There are several fluorescent reagents that can react with intracellular GSH and permit determining cellular levels of glutathione. Monobromobimane is the agent of choice for measuring GSH in human cells [101].

It has been shown that resin monomers such as TEGDMA and urethane dimethacrylate (UDMA) cause early and extensive glutathione depletion in human fibroblasts [21,102,103]. This event may significantly contribute to the cytotoxic potency of these monomers. Walther *et al.* studied the effect of antioxidative vitamins on the cytotoxicity of HEMA and TEGDMA and they found that the vitamins decrease toxic effects of the monomers assessed by GSH depletion. Although this finding supported their proposed mechanism of HEMA or TEGDMA toxicity based on radical metabolites [104], it has been reported that TEGDMA does not elevate reactive oxygen species levels in primary human fibroblasts [105]. Noda *et al.* reported that resin monomers act partly via oxidative stress by increasing GSH levels at sublethal concentrations but they do not affect the glutathione redox balance [106]. Lefeuvre *et al.* demonstrated that the mechanism of TEGDMA toxicity is based on interference with GSH and GSH Transferase P1 activity[107], lipid peroxidation and mitochondrial damage [108].

##### Heat-Shock Protein assay

Heat shock proteins (HSPs) are multifunctional proteins that are expressed when cells are exposed to stress and help to protect cells against stress. Measurement of intracellular stress proteins after exposure of the cells to materials has been introduced as a new method for cytotoxicity evaluation of dental materials [109]. Noda *et al.* found that HEMA and TEGDMA significantly suppress HSP-72 expression in heat-stressed THP-1 monocytes even at sublethal concentrations [110].

##### Apoptosis assays

Apoptosis (programmed cell death) can occur as a response to a cell injury due to a toxic substance. Apoptosis is distinct from necrosis in both the biochemical and the morphological changes that occur. Some scientists have focused on the type of cell death caused by toxic substances from resin-based dental materials [22,89,92,111,112]. Several methods have been developed to distinguish live cells from early and late apoptotic cells and from necrotic cells. These methods include: apoptosis assays using nucleic acid stains such as the comet assay (Single-Cell Gel Electrophoresis) to detect damaged DNA, apoptosis assays using Annexin V conjugates, assays based on protease activity such as Caspases, assays using mitochondrial stains, assays using free radical probes, assays using ion indicators, assays using esterase substrates, and an assay that measures the ATP: ADP ratio.

##### Other assays

A cell migration assay and Tenascin expression have been used by Theilig *et al.* to evaluate the biological effects of dental resin monomers on human fibroblasts and keratinocytes [63]. Hikage *et al.* used a colony formation assay to assess the cytotoxicity of bisphenol A glycerolate dimethacrylate (BisGMA) on cytochrome P450-producing cells [87]. Kaga *et al.* investigated the biochemical effect of the dental monomers on tyrosine phosphorylation of L929 cells *in vitro* [26].

A summary of different assay techniques, their mechanisms and relative merits are described in Table 1. The choice of the endpoint and the recording method depends on the required information. Normally in the first stage, simple methods based on membrane damage or cell viability and proliferation should be used. If in the latter stage of development more detailed information concerning the mechanism of the toxic action is needed, or if a special test method requires specific endpoints, more complicated methods based on cell function should be used [3]. Although these tests provide detailed information on biological interactions between the cells and test materials, it is often difficult to translate the severity of the biological response observed in the in *vitro* tests to clinical situation. Therefore, *in vivo* biocompatibility tests sometimes become necessary to obtain an accurate and comprehensive biological risk assessment.

**Table 1:** Summary of different biological assays used in cytotoxicity tests.

Biological assay	Mechanism	Advantages	Disadvantages
**MTT**	Mitochondrial dehydrogenase activity	Rapid and inexpensive	Toxic to the cells
**Alamar blue**	Chemical reduction of culture medium	Accurate, and non-toxic fluorometric/colorimetric method.	Expensive
**Neutral red**	Membrane damage (stains vital cells)	Non toxic substance	Less accurate than Alamar blue
**Propidium iodide**	Membrane damage (stains dead cells)	It is possible to measure dead cells	Less accurate than Alamar blue
**LDH**	Cell damage	Simple assay, provides additional information when used with other assays.	Poor dynamic range, lack of sensitivity
**BrdU**	Proliferation	Simple, rapid and inexpensive.	Less sensitive
**^3^H-thymidine**	Proliferation	Rapid and sensitive	Radioactive assay
**DNA measurement**	Proliferation	Non-radioactive, sensitive and robust.	None
**Protein content**	Proliferation	Easy, rapid and precise	None
**Inflammatory markers**	Inflammation indicators	Clinically relevant	Expensive and time-consuming tests.
**GSH**	Toxicity indicator	Provides additional information about the toxicity of materials.	Expensive and sophisticated.
**HSP**	Stress indicator	Provides additional information about the toxicity of materials.	Expensive and sophisticated.
**Apoptosis**	Cell injury	Sensitive and specific for apoptotic cells	Expensive and requires specific equipment

#### 2.1.2. *In vivo* tests

*In vivo* biocompatibility tests are performed inside a living organism. Animal tests are the most common type of *in vivo* tests.

##### Animal tests

In animal tests material is implanted into the body of an animal to evaluate local reactions to the material. In this type of test it is possible to examine many complex interactions between the biological system and the material, thus it is more relevant than *in vitro* tests. However, animal tests are expensive, and time consuming, it is difficult to control variables, and there are some ethical problems with the use of animals. Furthermore there are always questions about the suitability of an animal species to represent the human response [2,113].

##### Implantation testing

**Figure 3:**
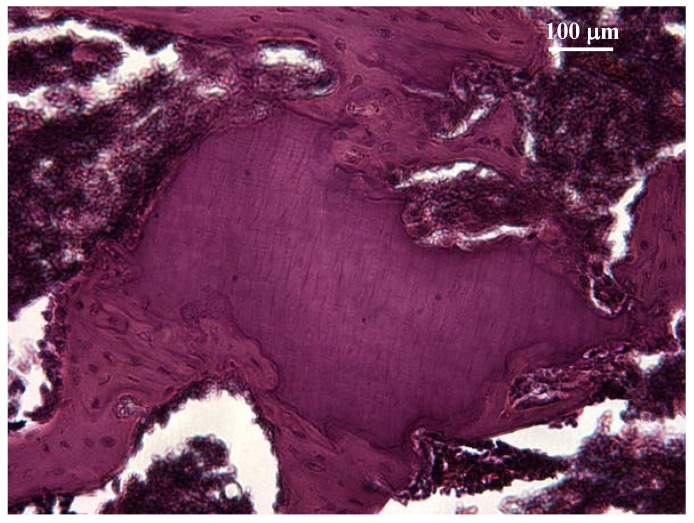
Processed dentine [124] fragment was implanted into a rat femur. Histological analysis after one month showed good biocompatibility and no sign of inflammation, necrosis, or fibrosis. The dentine has been completely incorporated into the newly formed bone.

In implantation studies, material specimens are implanted in the connective tissue [114,115], muscle [116], or into the bone [117] of an animal and an inert material such as silicone rubber serves as negative control. After a period of time the tissue reaction to test and control materials is examined (Figure 3). Biological variables include necrosis, inflammation, infiltration, fibrogenic cell function, and the organizational status of the resultant encapsulation [118].

Several studies have evaluated *in vivo* biocompatibility of different dental materials by implantation techniques [114,115,116,117,118,119].

##### Irritation test

In this test, materials are applied to the chorioallantoic membrane in fertilized hen eggs, and the membrane is examined by a photomacroscope for injury to the blood vessels. The average irritation score can be calculated from the recorded times for the debut of hemorrhage, lysis, and coagulation, and the test materials can be classified as being non, slight, moderate, or strong irritants, based on the irritation score. This technique has been frequently used for irritation testing of denture adhesives [120,121,122,123].

### 2.2. Pulpal toxicity testing

Pulpal reactions to resin-based dental materials can be assessed using various *in vitro* and *in vivo* test systems that are discussed in the following sections.

#### 2.2.1. *In vitro* pulpal toxicity

##### Monolayer cell cultures

Monolayer cultures of pulp cells and odontoblasts are suitable biological systems for the assessment of the biocompatibility of dentine bonding agents since the pulp tissue is the first target for toxic substances released from dentine bonding agents applied to deep cavities. Human and animal pulp cells [58,67,68,75], human THP-1 monocytes [36,99,110], and immortalized mouse odontoblast cell line MDPC-23 [125,126] have been used for biological assessment of dentine bonding agents and resin-based restorative materials. Thonemann *et al.* compared the response of L-929 mouse fibroblasts, primary and immortalized bovine dental papilla derived cell lines to dental resin components and reported that the ranking of the cytotoxic effects of the materials in the 4 cell types was identical but concentrations necessary for toxic response were different [64]. In clinical situation the pulp is normally protected by a layer of dentine and the components of the dentine bonding agents leach into pulp through the dentin tubules. To simulate the *in vivo* situation, 3D models of pulp tissue have been introduced.

##### Dentine barrier systems

Schmalz *et al.* [49] introduced an *in vitro* pulp chamber model for biocompatibility testing of dental materials. They modified a commercially available cell culture perfusion chamber by replacing the original membrane that serves as a substrate for cell growth by a dentine slice. Thus cell culture chamber was separated into two compartments by the dentine disc. The cell culture tissue was placed in one compartment and the test materials were introduced into the other compartment. So leachable components from the specimen could reach the cells through a dentin barrier.

Recently de Souza Costa *et al.* evaluated the transdentinal diffusion and subsequent cytotoxicity of self-etching adhesives on odontoblast-like cells using a similar dentine barrier system. It was shown that components from all investigated self-etching adhesive systems were able to diffuse through the dentin resulting in significant reduction of the cellular metabolism [127].

##### 3D tooth slice organ culture

In organ culture, parts of, or whole organs are maintained or grown in the culture medium so that their structure and function are preserved [128]. Since there is no blood circulation, oxygen transportation must be brought about by diffusion. Different organs have been used in the assessment of the biocompatibility of biomaterials such as mandibular first molar explants from mouse embryos [129], chick embryo femur [130] and embryonic chicken skin [131].

**Figure 4:**
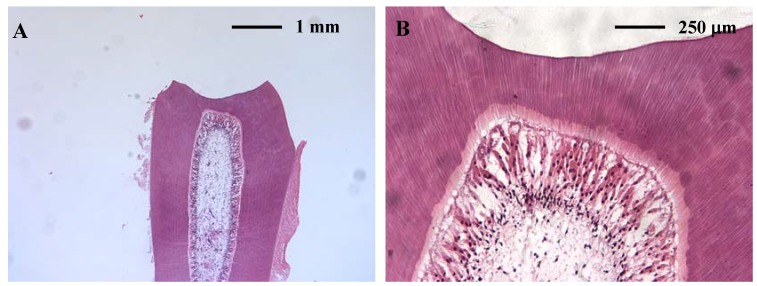
(A) Tooth slice organ culture. A cavity is prepared on the buccal side of the rat tooth to place the test materials; (B) viable odontoblasts and pulp fibroblasts are visible after 10 days organ culture.

Successful use of a rat tooth slice organ culture for the assessment of the cytotoxicity of dental materials on pulp cells has been reported, and it has been suggested that this method might have potential to replace some types of *in vivo* animal pulp tests (Figure 4) [132,133].

#### 2.2.2. *In vivo* pulpal toxicity

##### Animal and usage test

Usage tests are essentially clinical trials of a material. In these tests, the material is placed into a human volunteer in its final intended use. Sometimes monkeys may be used instead of humans. These tests are more clinically relevant than *in vitro* tests. However, they are expensive, time-consuming, difficult to control and interpret, and there are legal and ethical problems with these tests [2].

Usage tests for dentine-bonding agents have been documented [134]. Pulpal reactions to some dental adhesives [135,136,137], composite resin fillings [138,139,140], and resin-modified glass ionomer cements [141] have been examined by these tests.

In a recent study by de Souza Costa *et al.* biocompatibility of different resin-based materials was evaluated by applying the materials as liners in deep cavities prepared in sound human teeth. The teeth were extracted at different time points after the clinical procedures and were processed for histological evaluation. The results revealed that the materials tested had acceptable biocompatibility when applied in deep cavities [142]. However, in a similar previous study they reported that techniques for inlay cementation using distinct luting resin-based cements may cause specific pulpal damage [143].

## 3. Systemic Adverse Reactions

Systemic adverse reactions such as hypersensitivity and anaphylactic reactions associated with resin-based dental materials have been reported. Systemic adverse effects of resin-based materials can be assessed by four different tests: (1) Allergy testing, (2) systemic toxicity test, (3) estrogenicity test, and (4) genotoxicity tests.

### 3.1. Allergy tests

Allergy tests indicate a person’s allergic sensitivity to environmental substances. Commonly used allergy tests are skin tests, and patch test [144].

There are two forms of skin testing: percutaneous and intradermal. In percutaneous testing, allergen solutions are placed on the skin, and the skin is then either pricked with a needle or scratched, allowing the allergen to enter the skin. Intradermal testing involves directly injecting allergen solutions into the skin. In both tests, a reddened, swollen spot develops at the exposure site if the person is sensitive to the substance.

In the patch test the allergens are prepared in appropriate concentrations in white soft paraffin and are then spread on to discs, 1 cm diameter. The discs are placed on the skin, usually on the back and are kept in place for 48 hours. After 48 hours the discs are removed, and the skin is examined for any redness or swellings.

Allergic reactions to dental materials especially dental alloys [145,146,147,148] as well as resin-based dental materials [149,150,151,152,153] have been reported. These reactions are mainly denture stomatitis due to allergy to polymethyl methacrylate (PMMA) denture base material. Hypoallergenic denture base materials have been developed for patients with allergy to PMMA. These materials contain significantly lower residual monomer than PMMA [154].

### 3.2. Systemic toxicity

#### (a) Acute systemic toxicity

In a common method for this test, animals receive a series of injections for each material or extract and control. The animals are injected either intravenously or intraperitoneally and observed for seventy-two hours for systemic reactions. Studies on acute systemic toxicity of dental materials are rare. Culliton *et al.* developed histopathologic criteria to evaluate acute systemic toxicity of dental alloys [155]. They examined histopathologic changes in lung, kidney, and liver after 2 and 5 weeks and obtained conclusive and reproducible results.

#### (b) Chronic systemic toxicity

This test is designed to determine the harmful effects from multiple exposures to test materials or extracts during a period of 10% of the total life of the test animal.

### 3.3. Estrogenicity test

Chemicals can interact with steroid hormone receptors and affect human health by disrupting normal endocrine function [156]. There are several *in vitro* techniques to determine estrogenic activity of materials. These methods include the MCF-7 cell proliferation assay (E-Screen), receptor binding assay, and reporter gene assay using cell lines and yeast cells [157].

The E-Screen assay is based on the ability of MCF-7 breast cancer cells to proliferate in the presence of estrogens. This quantitative assay compares the cell number achieved by similar culturing of MCF-7 cells in the absence of estrogens (negative control) and in the presence of 17β-estradiol (positive control) and a range of concentrations of chemicals suspected to be estrogenic [158,159]. Olea *et al*. using this technique showed that BisGMA-based resins and sealants are estrogenic [160]. Later Schafer *et al.* confirmed the estrogenicity of Bisphenol A and BisGMA on MCF-7 cells [161].

The receptor binding assay determines the effect of the material on the number and affinities of binding sites of estrogen receptors [162].

The reporter gene assay is based on the ligand–dependent interaction of two proteins (a hormone receptor and a coactivator), and hormonal activity is detected by β-galactosidase activity. Mammalian cells and yeast cells can be used in the reporter gene assay. However, the yeast-based receptor assay is a sensitive, specific, and reproducible method for assessing chemical interaction with steroid receptors [163]. Several studies have demonstrated that some resin-based dental materials like fissure sealants, adhesives, and composites and their components, especially bisphenol-A related chemicals, are estrogenic in reporter gene assay [164,165,166,167].

### 3.4. Genotoxicity (Mutagenicity) test

Ames *et al.* introduced a mutation assay utilizing bacteria to detect carcinogens and mutagens [168], and later they described revised methods for Salmonella mutagenicity test [169] which is the most widely used biological test for genotoxicity. Mammalian cells such as V79 Chinese hamster lung fibroblasts [170,171,172], human gingival fibroblasts [173], L929 mouse fibroblasts [174], human lymphocytes [175], and parotid gland tissue cells [176] have also been used in genotoxicity assessment of dental materials. The biological endpoint in genotoxicity tests is changes in the DNA (point or gene mutations) or in the chromosomes themselves (chromosomal aberrations) [3].

Schweikl *et al.* showed that TEGDMA induces large DNA sequence deletions in Salmonella and V79 cells [177,178] and causes cell cycle delays through p53-dependent and independent pathways in various cell lines [179]. They also studied mutagenic activity of dentine bonding agents and found that only glutaraldehyde containing adhesives elicited a strong mutagenic effect in Salmonella and V79 cells [170,180,181]. However, it has been shown that extracts from dentine bonding agents including glutaraldehyde-free adhesives induce protooncogen expression in human gingival fibroblasts [173].

In the recent decade some studies have concentrated on the mutagenicity of epoxy resin monomers designed for the development of non-shrinking dental composites. These experiments have demonstrated that some of these monomers cause DNA damage and cell-cycle disruption in mammalian cells [174], and have mutagenic effects on *Salmonella typhimurium* [182]. However, Eick *et al*. showed that extracts from optimized oxirane/polyol dental composites were non-mutagenic in the Ames test [73]. In another study Kostoryz *et al.* found that expanding monomers with epoxy resin systems (spiroorthocarbonates) were non-mutagenic and suggested their potential use for development of biocompatible non-shrinking composites [183].

## 4. *In vitro* Biocompatibility Studies of Resin-based Dental Materials

### 4.1. Initial experiments

Resin-based dental materials include composite resins, dentine and enamel adhesives, compomers, resin-modified glass ionomer cements, and denture base materials. The direct filling acrylic materials were introduced in the early 1950s as a substitute for silicate cements for the direct restoration of anterior teeth. The composite resins that were developed by Dr. Raphael Bowen in 1962, consist of a polymer matrix (BisGMA) and a ceramic filler. Tronstad and Spangberg evaluated the *in vitro* biocompatibility of a methylmethacrylate composite material (Polycap), a conventional methyl-methacrylate resin (Sevriton), and a composite based on Bowen’s resin (Concise). The toxicity of Polycap was less than that of Sevriton and Concise, and Polycap’s toxicity reduced after setting [184]. The limitations of these early experiments were that tested materials were in their final product forms regardless of the diversity of ingredients used in their compositions. In other words, toxicity of the components of these materials was not assessed separately, and also component release from tested materials was not taken into the account.

### 4.2. Components of resin-based materials

Hanks *et al.* evaluated cytotoxic effects of 11 components of composite resins on cultured mammalian fibroblasts and found that ethoxylated bis-phenol A dimethacrylate was the most toxic molecule [29]. It has been found that TEGDMA and HEMA have significant time dependent toxicity on human and animal lung cells [91]. Geurtsen *et al.* examined the cytotoxicity of 35 dental resin composite monomers and additives in human and animal fibroblast cultures and found that the most toxic materials were: BisGMA, TEGDMA, UDMA, BisEMA, DEGDMA [41]. Thonemann *et al.* also showed that the ranking of material toxicity on primary and immortalized bovine pulp cell lines and L929 cells was: BisGMA > GMA > HDDM > BPA > CQ > TEGDMA > HEMA > MMA [64]. Ratanasathien *et al.* examined the cytotoxic interactive effects of the monomers used in dentine bonding agents on mouse fibroblasts and concluded that both exposure time and interactions between DBA components may be important parameters in determining the cytotoxicity of DBAs [31].

Rathbun *et al.* showed that after storing dental composite specimens in organic solvents, their toxicity on fibroblast cultures was decreased by 90 percent. They concluded that organic solvents remove the leachable toxic components from dental composites [30].

This fact inspired researchers to concentrate on toxic effects of the eluates from resin-based dental materials.

### 4.3. Eluates from resin-based materials

Lefebvre *et al.* examined the cytotoxicity of eluates from six different light-polymerized denture base resins by extracting leachable ingredients by culture medium. Components released from tested materials had prolonged toxic effects on hamster epithelial cells [70]. In another study they confirmed that even after polymerization, components used in dental resins may elute into the immediate environment and alter cell metabolic processes [185]. Pelka *et al.* evaluated the toxicity of eluates from composites and compomers on brine shrimp larvae as a sensitive organism. Their technique was quick and inexpensive but it had low sensitivity [71]. Hung and Chang evaluated the cytotoxicity of the eluates of two RM-GICs, a compomer and two composite resins on human pulp cells. Eluates from all materials were cytotoxic to pulp cells, especially Superfil composite [67]. These findings suggest that toxic components elute into aqueous environments. However, leachable components responsible for the toxicity of the eluates were not identified in these studies.

It has been shown that among resin monomers TEGDMA is the main component that elutes into an aqueous environment from dental composites [10,186,187] and we demonstrated that it can be extracted in quantities sufficient to be cytotoxic to primary human oral fibroblast cultures [12,65]. Geurtsen *et al.* reported that dentine bonding agents also release chemicals in aqueous media, some of which (TEGDMA and HEMA) are cytotoxic in fibroblast cell cultures [33]. Kaga *et al.* by evaluating the effects of Scotchbond multi-purpose and Liner bond 2 on tyrosine phosphorylation of L929 cells demonstrated that the cytotoxicity of the primers & adhesives correlated well with the amounts of either HEMA or TEGDMA eluted [26]. Stanislawski *et al.* investigated factors responsible for the cytotoxicity of five resin-modified glass ionomer cements (RM-GICs), one metal reinforced GIC, and a zinc-oxyphosphate cement on human pulp cells. The results of their study suggested that the principal compounds responsible for cytotoxicity are unpolymerized resin monomers in RM-GICs and Cu^2+^ and Ag^+^ in the M-GIC [68].

### 4.4. Effect of monomer structure on biocompatibility

Some studies have focused on the possibility of replacing toxic leachable components with safer and less-leachable monomers. Kostoryz *et al.* studied *in vitro* cytotoxicity of epoxy-based dental resins and their components on L929 fibroblasts using indirect agar diffusion assay and MTT assay. They concluded that by the addition of spiroorthocarbonates and polyols in the formulation of epoxy-based resins it might be possible to develop biocompatible non-shrinking dental composites [25]. In a different study Wan *et al.* compared the influence of hyperbranched multi-methacrylate (H-MMA)-based composites on proliferation of human gingival fibroblasts with BisGMA and TEGDMA based composites. H-MMA-based resins had similar cellular responses or proliferation to the Bis-GMA/TEGDMA-based resin systems, but had less free monomer release [19].

In order to study the relationships of monomer structures and cytotoxicity, Yoshii *et al.* evaluated the cytotoxic effects of thirty-nine acrylates and methacrylates on HeLa S3 cells. All acrylates were more toxic than corresponding methacrylates in their experiment [188].

### 4.5. Effects of fillers and additives on biocompatibility

Apart from monomer structure, different types of composite resins have been biologically assessed. Silva *et al.* examined the biological effects of Zirconia-Hydroxyapatite (ZHA) composites using a combination of *in vitro* (Agar diffusion test) and *in vivo* tests (Acute toxicity test and skin irritation test). They concluded that ZHA composites are not cytotoxic and do not cause skin irritation or systemic toxicity [59]. Franz *et al.* compared the cytotoxicity of packable and non-packable dental composites on L-929 cells in a direct contact format. Advanced composites showed similar or more severe cytotoxicity than non-packable composites and the toxicity of all materials increased when applied in a larger increment [27]. Biological effects of commercial core and flowable composite resins have also been evaluated. These materials cause severe toxicity on mouse fibroblasts, even worse than composite resins [35]. It has also been demonstrated that the change in the chemical structure of the composite and the variation in the ratio of filler and monomer have a significant effect on the element release and cytotoxicity level of the material. Flowable derivatives of the traditional composite resins are more cytotoxic than their standards except for Ormocer flowable material [189].

Geurtsen *et al.* regarded a photoinitiator as the prime cause for cytotoxic reactions evoked by a cement containing resin components [41]. Michelsen *et al.* found high amounts of a UV stabilizer (hydroxymethoxybenzophenone) eluting from resin-based dental materials [190] and Wada *et al.* found this substance to demonstrate estrogenic activity [167].

The effects of ascorbate and Trolox (6-hydroxy-2,5,7,8-tetramethylchroman-2-carboxylic acid, a water-soluble derivative of vitamin E) on the biocompatibility of composites, compomers, GIC, and RM-GIC materials have been investigated. It has been reported that Trolox reduces the cytotoxicity induced by these materials on human gingival fibroblasts. However, ascorbate increases the toxic effects in a dose related manner [66].

### 4.6. Long-term Biocompatibility of Resin-based Materials

Several studies have evaluated long-term cytotoxicity of resin-based dental materials. Schedle *et al.* assessed the cytotoxicity of six composites, a compomer, and several dental cements using L929 fibroblasts and showed that all tested materials were cytotoxic immediately after production and their toxic effects were reduced after different preincubation periods in most cases [24]. However, Wataha *et al.* in a study demonstrated that resin-based materials continue to release sufficient components to cause lethal effects or alter cellular function *in vitro* even after two weeks of aging in artificial saliva [191]. Bouillaguet *et al.* studied long-term cytotoxicity of smart restorative materials, ormocers (organically modified ceramics), and highly filled resin-based materials by aging the specimens for 24 h to 8 weeks in culture medium. The results of their experiment showed that aging significantly influenced the cytotoxicity. Aging reduced the cytotoxicity of the materials except for smart materials [34]. The reduced cytotoxicity can be due to the reduction in the rate of elution as demonstrated by Ferracane *et al.* that the elution of nearly all of the leachable components from dental composites was complete within the first 24-hour [9]. Smart materials have been developed as a strategy to minimize the adverse effects of polymerization shrinkage. The rationale for these materials has been based on the assumption that marginal gap formation resulting from polymerization shrinkage cannot be completely avoided clinically. Thus, these materials are formulated to release ions including fluoride under acidic conditions. Fluoride releasing materials have shown cariostatic properties and may affect bacterial metabolism under simulated cariogenic conditions *in vitro*. However, it is not proven by prospective clinical studies whether the incidence of secondary caries can be significantly reduced by the fluoride release of restorative materials [192].

Recently, the effect of curing method on cytotoxicity of composite resins has been assessed. In an experiment by Nalcaci *et al.* three different methods of light-curing (standard, soft start, and fast cure) were applied to three types of composite resins (flowable, condensable, and hybrid composites). There was no significant difference in cytotoxicity of the composite materials cured with different methods [193].

### 4.7. In vitro studies of dentine bonding agents

It has been shown that dentine bonding agents have toxic effects on immortalized odontoblast-like cells [125]. *In vitro* cytotoxicity of modern dentine adhesives has been assessed in a study by Szep *et al.* All tested materials caused cytotoxic effects of human gingival fibroblasts [20] Cytotoxicity of Syntac Sprint, Prime and Bond12, and Single Bond on human dental pulp cells has also been documented [75]. However, the biological response of monolayers cell cultures directly exposed to dentine bonding agents might be exaggerated and may not be clinically relevant. Schmalz *et al.* using a three-dimensional culture of bovine pulp derived cells in a dentine barrier test, assessed the cytotoxicity of low-pH dentine bonding agents (All-Bond 2, Prime and Bond, Syntac Single, Syntac Classic, and Prompt L-pop) and demonstrated that these materials do not show toxic reaction in this dentine barrier test [76]. Therefore they concluded that pulp damage caused by the tested materials is unlikely if a dentine layer protects the pulp.

### 4.8. Studies on the Mechanisms of Monomer Toxicity

Biological effects of resin monomers have been widely investigated using more sophisticated techniques especially in the recent decade. It has been shown that TEGDMA and HEMA can significantly modulate the expression of HSP72 in human THP-1 monocytes at sublethal concentrations, and the response depends on material, concentration and time after heat stress [110]. In another study sublethal exposure to TEGDMA and HEMA for two weeks suppressed LPS-induced TNF-α secretion from THP-1 monocytes [36]. Also it is proven that TEGDMA and HEMA are cytotoxic and apoptotic to human and animal cells in a dose and time dependent manner [22,111]. In order to discover the mechanism of TEGDMA-induced apoptosis, Spagnuolo *et al.* studied the apoptosis and necrosis induced by TEGDMA in human primary pulp cells. They found that inhibition of phosphatidylinositol 3-kinase (PI3K) amplified apoptosis caused by TEGDMA and Akt phosphorylation was inhibited in the presence of TEGDMA. They suggested that depression of PI3K signalling might be a primary target in TEGDMA-induced apoptosis [194]. The association of oxidative stress with the apoptosis and mutagenicity induced by the resin monomers has also been reported [195].

It has been reported that resin components can evoke either immunosuppression or immunostimulation on mitogen-driven proliferation of T-cells [196]. Theilig *et al.* studied the effects of Bis-GMA and TEGDMA on proliferation, migration, and tenascin expression of human fibroblasts and keratinocytes. They illustrated that Bis-GMA could affect migration of keratinocytes and altered the expression of the extracellular matrix component tenascin. Thus Bis-GMA may significantly influence the healing of injured oral tissues [63]. Bis-GMA has also been associated with high embryotoxicity and teratogenicity [197]. Although resin monomers are toxic to most human and animal cells, Hansel *et al.* reported that release of EGDMA and TEGDMA from resin composites stimulate the growth of the caries-associated micro-organisms [198]. However, Takahashi *et al.* demonstrated that the apparent biomass increase during incubation with ethyleneglycol monomers is not caused by promotion of bacterial proliferation, but by polymerization of resin monomers to form vesicular structures attached to cells [199].

Kostoryz *et al.* studied the biocompatibility of BisGMA, BFDGE, and their metabolites using three biocompatibility tests: cytotoxicity, Ames mutagenicity, and estrogenicity test. Hydroxylated metabolites were non-mutagenic, non-estrogenic, and less cytotoxic than their parent monomers [28]. Cytotoxicity of BisGMA and BPA on cytochrome P450 (CYP)-producing cells has also been examined. These monomers are not metabolically activated by CYP3A4 or CYP3A7 and they are neither activators nor inhibitors of CYP [87]. Issa *et al.* illustrated that resin monomers show a variety of toxic effects and they alter MTT and LDH activity in human gingival fibroblasts [23].

Although the details of the mechanisms leading to cell death, genotoxicity, and cell-cycle delay are not completely understood, resin monomers may be able to alter the functions of the cells of the oral cavity. Pathways regulating cellular homeostasis, dentinogenesis, or tissue repair may be modified by monomers at concentrations well below those which cause acute cytotoxicity [200].

## 5. *In Vitro* Versus *in Vivo* Tests

Reports on the biological safety profile of different resin-based dental materials show that there are contradictory data obtained from *in vitro* and *in vivo* studies. Usually, *in vitro* systems are more sensitive to test materials than the *in vivo* systems. The most efficient, cost-effective, and relevant way to ensure the biocompatibility of materials is to use a combination of *in vitro*, animal and usage tests [113]. The products are only submitted to *in vivo* tests when satisfactory results are obtained with *in vitro* tests [201]. However, no experimental study can guarantee 100% safety for any substance [202], therefore it is important to identify materials that have potential risk of adverse reactions to the patients when the materials are available on the market.

## 6. Post-Market Surveillance

Post-market surveillance is evidence-based gathering information about how safe the materials actually are. It serves as an early warning system to exclude materials that have potential risk of adverse reactions if their risk was not identified in pre-market tests. It can also reveal predisposing factors to specific adverse reaction and can compare side effects of similar products as well. Finally this system allows safety monitoring of a product during its use on the market.

The first national reporting system for adverse reaction to dental materials was established in Norway in 1993 [203]. The reporting procedure was based on voluntary spontaneous reporting by dentists and physicians. Reported reactions were compared with findings obtained by clinical examination of patients with suspected reactions to dental materials. From 1993 to 1999, a total of 899 reports were received and 253 patients were referred to clinical examination. According to this survey, a major source of confirmed adverse reactions was amalgam. Metals and resin-based materials were in second and third place, respectively [204].

A similar national reporting project was set up in Sweden in 1996. The aim of this survey was to clarify the nature and the incidence of side-effects associated with dental materials [205].

In 1999 the national survey of adverse reactions to dental materials in the UK was established in Sheffield [4]. In the adverse reaction reporting project (ARRP), green reporting forms were distributed to dental surgeries and laboratories in the UK. From 1999 to 2002, ARRP received 1,075 reports of suspected adverse reaction seen or experienced by dental staff and patients. The results of this study showed that, contact with acrylic resin was the main cause of hand dermatitis in dental technicians, and more than 12% of adverse reactions in patients were associated with resin-based dental materials.

The main limitation of the post-market surveillance is that adverse reactions can be under reported by the clinicians due to the lack of awareness and lack of clarity as to what constitutes an adverse reaction. Therefore, there is a need to raise the awareness among dental professionals of the potential for adverse reactions due to restorative dental materials.

## 7. Future Developments

Advanced resin-based restorative materials having the capability of repairing the discontinuities in the composite resin have been invented [206]. These materials are called self-healing composites. These composites contain unpolymerized monomers encapsulated in microspheres. When a fracture occurs, the microsphere is ruptured and the monomer fills the fracture and polymerizes. These materials provide increased resistance to fracturing, and thus remain substantially intact for a longer period of time, preserving the integrity of the restoration. Since they contain unpolimerized monomers, further *in vitro* and *in vivo* biocompatibility studies are necessary to ensure the safety of these new products.
